# Correlation of Physical Activity Level with Muscle Strength and Size During One Week of Knee Joint Immobilization

**DOI:** 10.3390/jfmk10020192

**Published:** 2025-05-27

**Authors:** Kylie K. Harmon, Zahra Pourhatami, Dylan Malinowski, Ryan M. Girts, Jonathan P. Beausejour, Jeremy S. Wydra, Joshua C. Carr, Jeanette Garcia, Matt S. Stock

**Affiliations:** 1 Department of Exercise Science, Syracuse University, Syracuse, NY 13244, USA; 2 Department of Health and Natural Sciences, Pfeiffer University, Misenheimer, NC 28109, USA; 3 School of Kinesiology and Rehabilitation Sciences, University of Central Florida, Orlando, FL 32816, USA; 4 Department of Kinesiology, Kansas State University, Manhattan, KS 66506, USA; 5 School of Sport Sciences, West Virginia University, Morgantown, WV 26506, USA

**Keywords:** disuse atrophy, muscle mass, rehabilitation, accelerometry, ultrasound

## Abstract

**Background**: Knee joint immobilization is common after surgery or injury. Whether remaining physically active during immobilization preserves muscle strength and size has not been studied. **Objectives**: This observational study examined correlations between muscle strength, size, and physical activity (PA) levels during one week of knee joint immobilization. **Methods**: Nine healthy adults (five males, four females) immobilized their left knee and ambulated with crutches for one week. Ankle accelerometers monitored compliance and tracked PA. Isometric and concentric isokinetic peak torque at 30°/s and 180°/s and vastus lateralis (VL) cross-sectional area (CSA) were assessed before and after immobilization. Bivariate correlations were used to examine relationships between time spent in sedentary, light, moderate, and vigorous PA, and changes in isometric and concentric isokinetic peak torque, as well as VL CSA. **Results**: After immobilization, isometric strength declined by 17.1%. Concentric isokinetic peak torque declined by 5.5% at 30°/s and 2.3% at 180°/s. VL CSA declined by 6.7%. There were weak correlations between strength measures and PA levels (*r* = −0.497–0.574; *p* = 0.106–0.709). For CSA, an unexpected pattern was found in which greater sedentary time was correlated with decreased atrophy (*r* = 0.701; *p* = 0.035), but light (*r* = −0.673; *p* = 0.047) and moderate (*r* = −0.738; *p* = 0.023) PA levels were correlated with increased atrophy. Vigorous PA had weak correlations with CSA (*r* = −0.321; *p* = 0.399). **Conclusions**: Contrary to our hypothesis, increased PA levels were not correlated with the preservation of strength and were correlated with greater declines in CSA during knee joint immobilization.

## 1. Introduction

Knee joint immobilization is a commonly used clinical intervention to promote healing after injury or orthopedic surgery. Short-term knee joint immobilization is effective in reducing pain and swelling, as well as protecting the tissues from further injury [1]. Despite this critical role in the healing process, immobilization is not without consequences. The deleterious effects of immobilization, such as muscle atrophy and strength loss, can make recovery challenging, extending rehabilitation timelines while simultaneously impairing activities of daily living and quality of life. As such, much attention has been given to the preservation of function throughout periods of immobilization [2,3].

Although physical activity (PA) is typically limited during immobilization to prevent further injury to the area and due to difficulty using prescribed mobility aids (i.e., crutches, braces), it is not prohibited entirely. Certain types of PA may be beneficial to maintain cardiovascular health, mitigate muscle atrophy, and prevent vascular complication [4,5,6]. In fact, many clinicians are more regularly recommending early mobilization/movement as soon as possible after certain orthopedic interventions (e.g., anterior cruciate ligament repair, total knee arthroplasty) [7]. Further, given the recent emphasis on the importance of daily step count, maintaining a minimum level of daily ambulation may have beneficial health effects [8]. Despite this, there is extremely limited evidence to indicate whether daily step count impacts immobilization-induced deficits. To the best of our knowledge, this has only been directly examined in a recent study which observed that 2000 steps per day (with no other added PA) were not sufficient to protect skeletal muscle health nor mitigate catabolic effects of prolonged physical inactivity in older adults [9]. While there have been a wide range of experimental efforts to preserve muscle size and strength during immobilization, the impact of daily habitual PA has not been thoroughly examined. Researchers utilizing experimental lower-limb immobilization/disuse models often track PA to monitor compliance but, to the best of our knowledge, have not directly investigated how PA levels may impact experimental outcomes [10,11,12,13,14]. If a link between PA, muscle strength, and muscle size of the immobilized limb exists, researchers and healthcare providers would be better able to provide guidance on daily PA for both research participants and clinical patients. Ultimately, there is little to no evidence on clear specific PA or step count thresholds that should be met to protect skeletal muscle health and function during periods of reduced activity due to limb immobilization.

Therefore, the purpose of this study was to examine PA levels (sedentary, light, moderate, and vigorous) during one week of knee joint immobilization and the correlation with vastus lateralis (VL) muscle strength and size. Given the known atrophic effects associated with mechanical unloading [2,3,15,16,17], we hypothesized that spending more time in sedentary activities and engaging in light PA would be linked to greater muscle weakness and atrophy compared to moderate and vigorous PA levels.

## 2. Materials and Methods

### 2.1. Study Design

This observational study was part of a larger randomized controlled trial investigating the effects of one week of knee joint immobilization on muscle strength, muscle size, and neuromuscular function [12], followed by an examination of the timeline to return to baseline levels of strength [18]. One week of immobilization was chosen based on previous investigations indicating that this timeline is sufficient to induce significant deficits in both muscle strength and size [2,3,19]. The left limb was chosen as the immobilized limb to allow participants to operate a motor vehicle as needed. Before data collection sessions, participants underwent a thorough familiarization visit to ensure comprehension of study protocols and minimize the influence of a learning effect. Torque data were collected during baseline testing (PRE) and one week later (POST). All visits to the laboratory occurred at the same time of day (±one h). Throughout the study, participants were asked to refrain from alcohol, keep their dietary habits consistent, and comply with the immobilization protocol. Participants were instructed to avoid all structured exercise, including both cardiovascular and resistive activities of the upper and lower body, during the immobilization week. However, they received no specific instructions on engaging in or limiting general PA. Participants were simply told to go about their daily lives as they normally would while complying with the immobilization protocol. To ensure safety and compliance, each participant was paired with a member of the research team, who served as their compliance officer. Each compliance officer checked in with their assigned participants daily and assisted with any concerns or issues that arose. Participants were compensated USD 200 for completion of the entire study, as well as a t-shirt. All protocols were approved by the University Institutional Review Board (STUDY00003289) and conformed to the standards set by the latest revisions of the Declaration of Helsinki. This study was prospectively registered on ClinicalTrials.gov (Identifier: NCT05072652).

### 2.2. Participants

A target sample of healthy adults was recruited from the university community. Nine healthy, college aged adults (5 males, mean ± SD age = 22 ± 4 years, height = 180.5 ± 6.6 cm, mass = 80.7 ± 5.0 kg; 4 females, mean ± SD age = 20 ± 1 year, height = 158.5 ± 5.1 cm; mass = 55.8 ± 5.3 kg) completed all study procedures and were included in the final analyses. Prior to study enrollment, participants were required to complete a detailed health history questionnaire to ensure no contraindications to participation. Exclusion criteria included body mass index (BMI) below 18.5 or above 35.0 kg/m^2^, musculoskeletal issues (i.e., back, shoulder, or knee pain) that may have interfered with the use of crutches, recent history of surgery on the hip or knee joints, family history of thrombosis, history of seizures or fainting, use of certain medications (e.g., muscle relaxants or benzodiazepines), use of a cardiac pacemaker, or current pregnancy. Given previous investigations indicating that training status does not impact the magnitude of disuse-induced deficits [20,21,22], both trained and untrained participants were enrolled. All participants were notified of study risks and completed an informed consent document prior to enrollment.

### 2.3. Anthropometrics and Ultrasonography

During the first laboratory visit, body mass and height were assessed using a physician’s scale and stadiometer (Seca 700, 0.2–250 kg weight capacity, 0.1 kg gradation, 122–230 cm height range, 1 mm gradation, Chino, CA, USA), respectively. Ultrasonography images of the VL muscle were taken with a portable B-mode imaging device (GE Logiq BT12, GE Healthcare, Milwaukee, WI, USA) and a multi-frequency linear-array probe (12 L-RS, 5–13 MHz, 38.4 mm field of view; GE Healthcare, Milwaukee, WI, USA). Prior to image capture, participants rested supine for five minutes to allow the redistribution of fluids from their quadriceps muscles [23]. The panoramic function (LogiqView, GE Healthcare, Milwaukee, WI, USA) was used to obtain images of VL in the transverse plane. Measurements were taken at 50% of the straight-line distance between the greater trochanter and the lateral epicondyle of the femur. A high-density foam pad was secured around the thigh with an adjustable strap and the probe was moved along it to ensure desired probe movement in the transverse plane. Ultrasonography settings (frequency: 12 MHz, gain: 55 dB, dynamic range: 72) were kept consistent across participants. To ensure optimal image clarity, a standardized depth of 5.0 cm was utilized [24] and kept consistent across trials. A generous amount of water-soluble transmission gel (Aquasonic 100 ultrasonography transmission gel, Parker Laboratories, Inc., Fairfield, NJ, USA) was applied to the skin to allow for immersion of the probe surface during measurement to enhance acoustic coupling. Three images of the muscle were obtained, with mean values being used for statistical analyses.

The ultrasonography images were digitized and examined with ImageJ software (version 1.46, National Institutes of Health, Bethesda, MD, USA) at the conclusion of the study. The polygon function was used to outline the borders of the transverse images of the VL. After scaling the image units from pixels to cm, muscle cross-sectional area (CSA) (cm^2^) was determined with the polygon function. The same experienced researcher obtained and analyzed all ultrasound images [25] and demonstrated excellent interrater reliability (ICC [3,1] = 0.987, SEM = 0.68%).

### 2.4. Assessment of Isometric and Isokinetic Concentric Torque

All isometric and concentric strength measurements were performed with the left (immobilized) knee extensors via a Biodex System 4 isokinetic dynamometer (Biodex Medical Systems, Shirley, NY, USA). Before testing, participants were seated in the dynamometer with restraining straps placed over the trunk, pelvis, and thigh. The input axis of the dynamometer was aligned with the axis of rotation of the knee. Each participant’s dynamometer chair settings were recorded and replicated during subsequent testing sessions. The chair was adjusted so that isometric torque testing was performed at hip and knee joint angles of 90°. The lower leg was secured to an anti-shear attachment with the pad placed over the tibialis anterior, just superior to the malleoli. Prior to testing, participants were given a warm-up consisting of three 10 s contractions at 50%, one 5 s contraction at 75%, and one 3 s contraction at 90% of their perceived maximal torque.

After the warm-up, participants performed three 5 s maximal voluntary contractions (MVCs) of the knee extensors. Participants were instructed to push as hard and fast as possible. During the MVC, participants received visual feedback of their torque output on a screen placed at eye-level ~1.5 m in front of them, as well as strong verbal encouragement from the research team. The highest recorded value was designated as the MVC peak torque value (Newton meters; Nm).

Following the assessment of isometric torque, participants performed dynamic strength tests of the left knee extensors on the Biodex isokinetic dynamometer. Specifically, five repetitions each of concentric isokinetic muscle actions at 30°/s and 180°/s were performed with at least two minutes of rest between each velocity. Participants were encouraged to push hard and fast throughout the full range of motion. Isometric and concentric isokinetic peak torque values were analyzed with custom LabVIEW software (version 20.0, National Instruments, Austin, TX, USA) by visually determining the repetition with the highest peak torque (Nm), which was used for further analysis.

### 2.5. Immobilization Procedures

At the end of PRE, participants assigned to immobilization groups were fitted with a knee joint immobilization brace (T Scope ^®^ Premier Post-Op Knee Brace, Breg, Inc., Carlsbad, CA, USA). The brace was locked at 90° of knee flexion, to ensure that the foot was raised off the ground. This position prevented normal weight-bearing and allowed the knee extensors to stay relaxed. Participants were instructed to only remove the brace in bed, prior to sleep. During bathing, participants were instructed not to remove the brace, but to keep it dry by covering it with a large plastic bag provided by the research team. For their safety and comfort while bathing, each participant was offered a shower chair (Medline Shower Chair Bath Seat with Padded Armrests and Back, Medline Industries, Inc., Northfield, IL, USA). For ambulation, participants were provided with axillary crutches (Cardinal Health Axillary Crutch, Adult, Adjustable, Cardinal Health, Inc., Dublin, OH, USA). Participants were fitted with and trained in the proper use of crutches, including navigation of stairs, doors, and other community obstacles. Participants were given a stocking (McKesson Tubular Cotton Stockinettes, San Francisco, CA, USA) to wear underneath the brace, which was measured to extend from the proximal thigh to the ankle. The stocking was intended to mitigate discomfort and minimize the risk of skin irritation from the brace. This was worn at all times and only removed during sleep when the brace was removed. A compression stocking (Medi-Pak Anti-Embolism Stockings, McKesson, San Francisco, CA, USA) was provided to be worn while sleeping, to reduce the risk of blood clots. In accordance with previous knee joint immobilization studies [10,11,20,26,27], participants performed twice daily (morning and evening) range of motion movements of the ankle and knee to minimize the risk of vascular and muscular issues due to immobilization. Movements were performed while lying in bed, and consisted of knee flexion, ankle pumps, and passive leg lowers. A video providing instructions was provided to participants.

### 2.6. Measurement of Compliance and Physical Activity

To ensure compliance with the immobilization protocol, participants were fitted with Actigraph GTX9 accelerometers (ActiGraph Inc., Pensacola, FL, USA) around both ankles. Participants were instructed to wear the accelerometers for the entire duration of the immobilization week, to be removed only when showering or bathing. Accelerometer data monitored overall wear-time compliance utilizing criteria established by Troiano [28] and compliance with the immobilization protocol by examining differences in PA intensity and step counts between the right (ambulatory) and left (immobilized) leg [14]. Wear-time compliance required that participants wear the device for a minimum of 4 days (10 h per day) over the 7-day immobilization period [28]. Non-wear time was defined by an interval of at least 60 consecutive minutes of zero activity intensity counts, with allowance for 1–2 min of activity intensity counts between 0 and 100 [29]. To determine PA levels during immobilization, assessment of counts per minute (CPMs) of the right leg were utilized [28]. Briefly, mean CPM were determined by evaluating raw accelerometer data without imposition of any criteria other than wear and non-wear time. Mean CPM were then calculated by dividing the sum of activity counts for a valid day by the number of minutes of wear time in that day across all valid days. This methodology allowed for four levels of PA to be established: sedentary, light, moderate, and vigorous. PA levels were established as follows: sedentary = 0–99 CPM, light = 100–2019 CPM, moderate = 2020–5998 CPM, and vigorous ≥5999 CPM. Time spent in activity of a defined intensity was determined by summing minutes per day where the count met the criterion for that intensity. For greater detail, the reader is directed to the work of Troiano [28].

### 2.7. Nutrition Tracking

Throughout the immobilization week, participants completed a food and hydration log tracking three days of intake: two weekdays and one weekend day. They were shown examples of what qualified as sufficient and insufficient tracking, and were instructed to be as detailed as possible, including brand names, preparation methods, and amounts consumed. When food entries were not sufficiently specific, well known national brands were prioritized and a standard single serving size was used. The same member of the research team used a nutrition tracking app (MyFitnessPal, version 22.7.5.39110, My Fitness Pal, Inc., Austin, TX, USA) to sum daily calorie and macronutrient intake. The mean calorie and macronutrient consumption of the three days was used for analysis.

### 2.8. Statistical Analyses

Prior to further statistical analyses, variables were checked for normal distribution with the Shapiro–Wilk test. PRE to POST changes were examined with Student’s *t*-tests for paired samples. Cohen’s *d* effect sizes were used to highlight important pairwise differences, with values of 0.2, 0.5, and 0.8 corresponding to small, medium, and large effects, respectively [30]. Pearson product moment correlations (*r*) were used to determine the relationship between VL CSA, MVC peak torque, concentric isokinetic peak torque at 30°/s and 180°/s, and PA levels (sedentary, light, moderate, and vigorous). Correlation coefficients of *r* = 0.10, 0.30, and 0.50 represented weak, moderate, and strong correlations, respectively [30]. All variables are represented as percent change from the pre- to post-test and are shown as mean ± standard deviation (SD). An alpha level of 0.05 was used to determine statistical significance. JASP software (version 0.18.1, University of Amsterdam, Amsterdam, The Netherlands) was used for all statistical analyses.

## 3. Results

### 3.1. Compliance and Physical Activity

All nine participants had at least 4 days of accelerometer data for a minimum of 10 h and were considered compliant in accordance with Cook et al. [14]. The average number of days worn was 6.89. There was a significantly higher number of steps per day (*p* = 0.004) on the right leg compared to the left (immobilized leg). The mean ± SD (range) daily step count for the right (ambulatory) leg was 3661 ± 1490 (1631–6600) steps.

**Figure 1:**
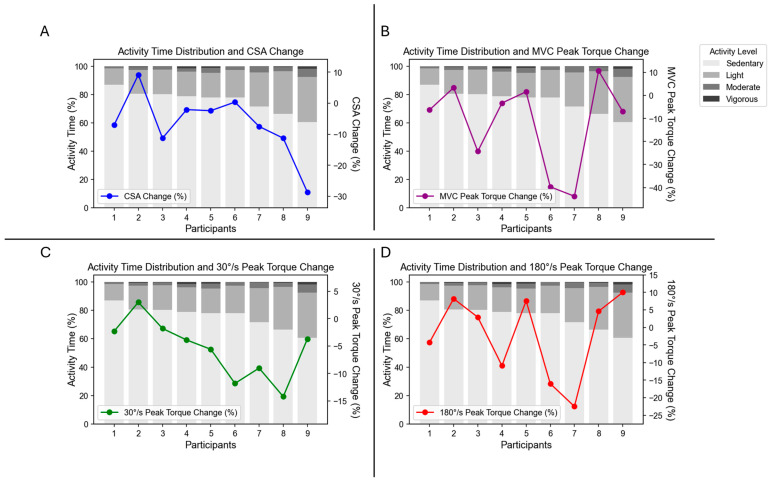
Individual participant data representing percent change in (**A**) vastus lateralis (VL) cross-sectional area (CSA), (**B**) maximal voluntary contraction (MVC) peak torque, (**C**) concentric peak torque at 30°/s, and (**D**) 180°/s, and time spent in specific physical activity (PA) intensity levels.

The mean ± SD percentage of daily time spent engaging in specific PA levels is displayed in Table 1. Individual participant PA data are displayed in Figure 1.

### 3.2. Muscle Strength and Size

Following one week of knee joint immobilization, isometric MVC peak torque declined by 17.14 ± 18.61% (*p* = 0.039; Cohen’s *d* = 0.820). Isokinetic concentric peak torque declined by 5.48 ± 5.36% (*p* = 0.022; Cohen’s *d* = 0.947) at 30°/s and by 2.27 ± 11.77% (*p* = 0.587; Cohen’s *d* = 0.189) at 180°/s. VL CSA declined by 6.72 ± 10.41% (*p* = 0.062; Cohen’s *d* = 0.724). PRE to POST changes are presented in Table 2.

### 3.3. Correlation with Physical Activity Levels

**Figure 2:**
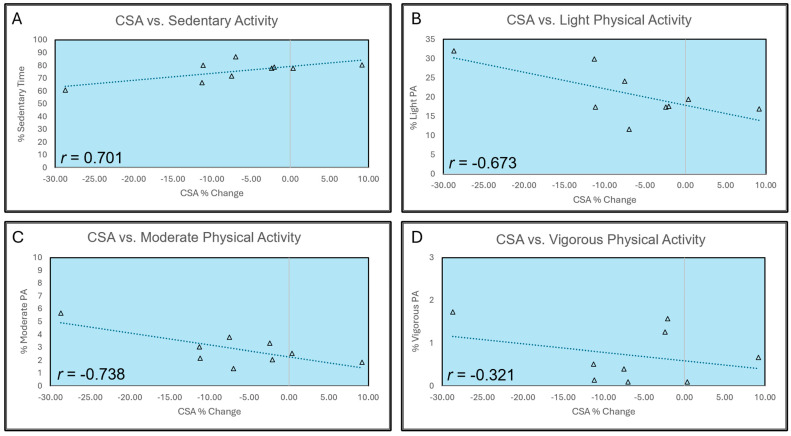
Individual participant data and correlations between vastus lateralis (VL) cross-sectional area (CSA) and time spent in (**A**) sedentary activity, (**B**) light physical activity (PA), (**C**) moderate PA, and (**D**) vigorous PA.

Changes in muscle strength demonstrated poor correlations with PA levels, as depicted in Table 3. Changes in VL CSA were positively correlated with sedentary activity (*r* = 0.701, *p* = 0.035) and negatively correlated with light (*r* = −0.673; *p* = 0.047) and moderate (*r* = −0.738, *p* = 0.023) activity. There was a weak negative correlation between changes in VL CSA and vigorous PA (*r* = −0.321, *p* = 0.399). Individual participant changes in MVC peak torque, concentric peak torque at 30°/s and 180°/s, VL CSA, and time spent in each PA level are presented in Figure 1. A graphical representation of correlations between changes in VL CSA and time spent at each PA level is presented in Figure 2.

### 3.4. Nutritional Intake

The mean ± SD (range) daily calorie intake was 1888.0 ± 415.1 (1251.0–2492.7) calories. The mean ± SD (range) of daily fat intake was 70.1 ± 20.8 (34.7–100.7) g. The mean ± SD (range) of daily carbohydrate intake was 206.0 ± 53.4 (161.7–305.0) g. The mean ± SD (range) of daily protein intake was 104.1 ± 31.0 (70.7–160.0) g.

## 4. Discussion

We sought to determine if disuse-induced deficits in muscle strength and size during one week of knee joint immobilization were mitigated by increased time spent engaged in higher levels of PA. Contrary to our hypotheses, changes in muscle strength were poorly correlated with all PA levels. Surprisingly, participants who engaged in greater amounts of light to moderate PA experienced greater decreases in VL CSA. However, participants who had high levels of sedentary activity did not experience decreases in VL CSA, nor did those who engaged in vigorous PA. Although unexpected, there are likely several compelling reasons for these findings.

One week of unilateral knee joint immobilization did result in appreciable decrements in muscle strength and size, with a 17.1% and 6.7% decrease in MVC peak torque and VL CSA, respectively. Although the decrease in VL CSA did not reach statistical significance, this may have been due to limitations of our sample size, as we did observe a medium–large effect (*p* = 0.062, Cohen’s *d* = 0.724). This is in line with previous research indicating that one week of lower-limb disuse is sufficient to induce losses in muscle strength and size [2,3,19]. However, to the best of our knowledge, no investigation has specifically examined whether PA levels play a mitigating role in immobilization-induced deficits. Recent work by Arentson-Lantz et al. [9] investigated whether meeting the minimum recommended PA threshold of 150 min per week of moderate intensity exercise was sufficient to counteract losses in leg muscle strength and mass in older adults undergoing bedrest. Participants performed blocks of ~2000 steps per day throughout 7 days of bed rest to meet the required threshold of 150 min of activity per week. However, this was not sufficient to preserve leg strength or leg lean mass. Like the participants in this study, those in Arentson-Lantz et al. [9] were otherwise healthy and subjected to experimental disuse. However, unlike the healthy, college-aged participants herein, those in Arentson-Lantz et al. [9] were older adults. Therefore, it is possible that the numerous deficits in musculoskeletal health caused by natural aging may have impacted their findings, which may differ in younger populations. Alterations to both the central (e.g., motor unit remodeling, decreased voluntary activation, decreased excitability) and peripheral (e.g., reduced muscle fiber size and number, alterations in excitation–contraction coupling, increased intramuscular adipose infiltration) systems occur with age, exacerbating the deficits incurred during disuse and making direct comparison between younger and older adults challenging [31,32]. Further, bed rest may result in greater systemic effects than unilateral limb immobilization, including hormonal changes, systemic inflammation, generalized muscle atrophy, and full-body insulin resistance, the magnitude of which likely requires a greater PA stimulus to overcome [33]. Still, it is clear that more PA was not a ‘better’ or stronger stimulus for offering protective effects against strength loss or muscle atrophy herein.

Although this study was not designed to test the effects of cross-education, in examining our findings, it may be important to consider the cross-education literature. Briefly, cross-education is a phenomenon in which unilateral strength training produces performance improvement in the untrained limb [34,35,36,37]. While the mechanisms of cross-education are not yet fully understood, it has been explored in immobilization models for its potential to preserve muscle strength and size, with promising findings [38,39,40,41]. However, two discrepancies exist between true cross-education investigations and the present study. First, to the best of our knowledge, the majority of current investigations on cross-education during immobilization are confined to the upper limbs, with limited previous investigation into the impact of cross-education in a lower-limb immobilization model. To our knowledge, only one study has investigated cross-education during lower-limb immobilization, finding no difference in quadriceps strength loss between the training and control groups [42]. Second, cross-education typically involves true strength training interventions, with routine activation of the contralateral target muscles via moderate-to-high intensity muscular contractions. Given that strength training was not performed herein, it is difficult to directly compare this intervention with the cross-education literature. Still, it is interesting to note that greater levels of routine muscular activation of the contralateral limb (albeit only during walking) did not result in protective effects of the immobilized limb. However, some evidence does suggest that lower-limb muscles are more susceptive to unloading than upper-limb muscles, particularly via peripheral mechanisms. A recent review by Campbell et al. [2] notes that in many experimental immobilization investigations, it appears that there is a greater influence of central neuromuscular function in the upper limbs, whereas the lower limbs are subject to greater impact from peripheral mechanisms. For example, during immobilization, the rate of atrophy in the lower-limb muscles is nearly double that of upper-limb muscles, which coincides with greater deterioration of contractile function and strength [2,43]. Since cross-education has primarily been studied in the context of upper-limb immobilization, it is possible that the central mechanisms in the upper limb are more responsive to cross-education interventions. In contrast, the muscles of the lower limb may be less affected by these interventions. Therefore, more research is needed to explore the effects of cross-education on lower-limb muscles to better understand their potential for adaptation.

Although the negative correlation between VL CSA and light and moderate PA levels was initially confounding, we believe there may be several possible reasons for these findings. Immobilization results in a cascade of catabolic processes, such as alterations in muscle protein synthesis and breakdown, increased reactive oxygen species (ROS) production, and increased inflammation [44]. Impairments in both fasting and post-prandial muscle protein synthesis have been observed during disuse, aiding in the observation of pronounced muscle atrophy [45,46,47]. This is further exacerbated by anabolic resistance during disuse, which results in a dampened protein synthetic response to increased amino acid availability [48]. Increased atrogin-1 and MuRF1 mRNA expression have been observed during immobilization, both of which play key roles in the ubiquitin–proteasome pathway thought to regulate muscle protein breakdown [15,44,49,50]. When a muscle ceases to contract, as it does during limb immobilization, FoxO3, a member of the FoxO family of transcription factors, is dephosphorylated, increasing the activity of atrogin-1 and MuRF-1, thereby activating the process of protein degradation [51,52]. Immobilization also damages mitochondrial membranes, resulting in increased ROS generation and oxidative stress [44,53]. Finally, immobilization can activate NFκβ signaling and increase the production of pro-inflammatory cytokines, further exacerbating ROS production [44,54]. This combination of factors during limb immobilization results in a particularly catabolic state characterized by rapid reduction in muscle size.

In general, PA helps to reverse or mitigate this process, with remobilization or resistive rehabilitation programs resulting in the regain of strength and/or mass [18,22,44]. However, given the combination of these detrimental processes, VL CSA may have been better preserved in the sedentary group due to less overall metabolic cost. It is possible that the additional PA of some participants increased metabolic cost to the point of greater atrophy due to increased metabolic demands combined with the cellular and molecular alterations discussed above. Further, it has previously been established that the energy cost of walking with crutches versus normal walking is nearly twice as great [55]. Anecdotally, many of our participants remarked on the difficulty of walking with crutches. It is possible that the combination of increased metabolic cost of crutches plus the increased metabolic expenditure due to greater levels of PA resulted in a hypercatabolic environment, with multiple factors contributing to protein breakdown, combined with decreased protein synthesis observed during immobilization. While it seems that this would certainly be the case for vigorous PA as well, our participants engaged in very little vigorous PA (Figure 1), resulting in less than 1% of their time on average (Table 1). It is likely that the amount of vigorous PA was so minimal that it did not result in appreciable correlation with CSA change.

Further exacerbating the possibility of catabolism in our participants, nutritional intake was likely insufficient to counteract the compounding effects of immobilization and increased metabolic expenditure. Negative energy balance can increase the loss of muscle mass and strength, which was likely the case for several of our participants [56]. While participants were instructed to keep their diet habits consistent, their habitual intake likely did not account for the increase in caloric needs due to the use of crutches, activity level, or stress on the body due to immobilization [56]. This subpar energy consumption likely added to atrophic effects observed in our more active participants. Protein intake was also of some concern in our participants. While they largely met the 1.4 to 2.0 g of protein per kg of body mass per day that is recommended to maintain a neutral nitrogen protein balance in healthy adults [57], the amount of protein needed to stimulate protein synthesis likely increases during immobilization due to anabolic resistance [45,46,56,58,59]. Indeed, muscle protein synthesis has been observed to be reduced by 50–60% in disuse models [60]. To attenuate muscle protein degradation during immobilization, protein intakes of closer to 2.0 to 3.0 g of protein per kg of body mass are recommended [57,61]. Only one of our participants met these recommendations, at 2.0 g of protein per kg of body mass. This subpar nutritional intake combined with the increased metabolic expenditure due to the use of crutches and greater PA may have resulted in an enhanced atrophic response.

Another possible reason for our findings may be related to our participants’ habitual PA levels. While we did not explicitly track PA before or after the immobilization intervention, many of our participants were undergraduates in a health and exercise science program. Many reported being recreationally active, regularly performing cardiovascular and resistance training. It is possible that the lack of mechanical loading was only novel for those who were habitually more active, whereas those who were sedentary were accustomed to low levels of PA. This may have resulted in less pronounced CSA changes in the sedentary group, as there was less overall change to their daily activity. Similarly, it is possible that those who did routinely engage in higher levels of PA were still engaging in lower overall PA than they were accustomed to, resulting in more measurable atrophy. However, as we did not explicitly track PA data prior to immobilization, these hypotheses are speculative and further research is needed.

This study had several limitations that need to be considered in interpreting the results. First, our sample size was small and included both males and females. Current evidence suggests differences in how men and women respond to immobilization, with most of the evidence suggesting that females experience more pronounced deficits in strength and size following knee joint immobilization [18,27,62,63]. Given our small sample size and primary research question, we elected not to analyze data from men and women separately. However, future investigations should further explore potential sex differences in how PA during immobilization impacts muscle strength and size. Additionally, as previously mentioned, we did not track habitual PA levels pre- or post-immobilization. While the results of our investigation indicate that greater levels of PA during immobilization resulted in no preservation of strength and greater muscle atrophy, to interpret these data fully, we need additional information on the routine habits of the participants undergoing immobilization. Future investigations would benefit from examining how changes in PA impact muscular outcomes, rather than the level of PA intensity during immobilization. Nutritional considerations are also a concern, as intake may have been subpar for the preservation of strength and mass, and we did not control intake but only gauged it via three-day self-report logs. Finally, the results of our experimental immobilization model likely differ from immobilization with associated trauma. The physiological response that occurs with trauma (e.g., injury or surgery) results in a variety of processes that further exacerbate losses in muscle strength and size [64,65]. Therefore, our findings may differ in true patient populations.

## 5. Conclusions

In conclusion, engaging in additional light and moderate PA does not appear to mitigate muscle atrophy or strength loss, and may even negatively impact the muscle CSA of the affected limb. Practitioners and clinicians working with patients recovering from injury or surgery should focus on interventions other than habitual PA to address immobilization-induced deficits. Individuals can engage in their preferred PA patterns during short-term immobilization without a significant impact on strength or CSA beyond what is typically incurred during disuse. Although the study provides novel insights, the results should be interpreted cautiously due to the small sample size, reliance on self-reported dietary data, and absence of baseline PA profiling.

## Figures and Tables

**Table 1:** Mean ± standard deviation (SD) percent of time spent engaging in specific physical activity (PA) levels.

PA Level	Percent of Time
Sedentary	75.68 ± 8.04%
Light PA	20.73 ± 6.63%
Moderate PA	2.87 ± 1.30%
Vigorous PA	0.72 ± 0.64%

**Table 2:** PRE to POST values [mean ± standard deviation (SD)]. MVC = maximal voluntary contraction; Nm = Newton meters; cm = centimeters. Effect sizes reported as Cohen’s *d*.

	PRE	POST	*p*-Value (Effect Size)
MVC peak torque (Nm)	183.58 ± 60.19	160.23 ± 56.34	0.039 (0.820)
Concentric peak torque @ 30°/s (Nm)	149.80 ± 36.40	142.34 ± 35.31	0.022 (0.947)
Concentric peak torque @ 180°/s (Nm)	106.23 ± 35.90	104.06 ± 32.55	0.587 (0.189)
VL CSA (cm^2^)	25.68 ±7.90	24.57 ± 8.36	0.062 (0.724)

**Table 3:** Pearson *r* and *p* values for bivariate correlations between changes in strength variables and physical activity levels. MVC = maximal voluntary contraction.

Pearson *r* (*p*)	Sedentary	Light	Moderate	Vigorous
MVC peak torque	0.286 (0.456)	−0.373 (0.323)	−0.146 (0.709)	0.574 (0.106)
Concentric peak torque @ 30°/s	0.440 (0.236)	−0.497 (0.173)	−0.275 (0.474)	0.197 (0.611)
Concentric peak torque @ 180°/s	−0.197 (0.611)	0.170 (0.661)	0.174 (0.654)	0.359 (0.343)

## Data Availability

Data generated or analyzed during this study are available from the corresponding author upon reasonable request.

## Abbreviations

The following abbreviations are used in this manuscript:

PA	Physical activity
MVC	Maximal voluntary contraction
CSA	Cross-sectional area
VL	Vastus lateralis
CPM	Counts per minute
